# Drug Prescription Profiles in Patients with Polypharmacy in Spain: A Large-Scale Pharmacoepidemiologic Study Using Real-World Data

**DOI:** 10.3390/ijerph18094754

**Published:** 2021-04-29

**Authors:** Miguel Ángel Hernández-Rodríguez, Ermengol Sempere-Verdú, Caterina Vicens-Caldentey, Francisca González-Rubio, Félix Miguel-García, Vicente Palop-Larrea, Ramón Orueta-Sánchez, Óscar Esteban-Jiménez, Mara Sempere-Manuel, María Pilar Arroyo-Aniés, Buenaventura Fernández-San José, José Ignacio de Juan-Roldán, Ignatios Ioakeim-Skoufa

**Affiliations:** 1 Drug Utilization Work Group, Spanish Society of Family and Community Medicine (semFYC), ES-08009 Barcelona, Spain; meresempere@gmail.com (E.S.-V.); caterinavicens@gmail.com (C.V.-C.); franciscagonzalezrubio@gmail.com (F.G.-R.); rpcsc@yahoo.es (F.M.-G.); vicentepaloplarrea@gmail.com (V.P.-L.); roruetas@gmail.com (R.O.-S.); oscarej@hotmail.com (Ó.E.-J.); marasemperemanuel@gmail.com (M.S.-M.); pilar.arroyo.anies@cfnavarra.es (M.P.A.-A.); venturafsj@gmail.com (B.F.-S.J.); dejuanroldan@gmail.com (J.I.d.J.-R.); 2 Dirección del Servicio Canario de la Salud, Plan de Salud de Canarias, ES-38004 Santa Cruz de Tenerife, Spain; 3 Centro de Salud de Paterna, Conselleria de Sanitat Universal i Salut Pública, Generalitat Valenciana, ES-46980 Valencia, Spain; 4 Centro de Salud Son Serra-La Vileta, Servicio de Salud de las Islas Baleares Ib-salut, ES-07013 Palma, Spain; 5 Institut d’Investigació Sanitaria Illes Balears (IDISBA), Servicio de Salud de las Islas Baleares Ib-salut, ES-07120 Palma de Mallorca, Spain; 6 Centro de Salud Delicias Sur, Servicio Aragonés de Salud, ES-50009 Zaragoza, Spain; 7 EpiChron Research Group on Chronic Diseases, Aragon Health Research Institute (IIS Aragón), ES-50009 Zaragoza, Spain; 8 Ministerio de Sanidad, Consumo y Bienestar Social, ES-28014 Madrid, Spain; 9 Hospital de Denia. Marina Salud, Conselleria de Sanitat Universal i Salut Pública, Generalitat Valenciana, ES-03700 Alicante, Spain; 10 Centro de Salud de Sillería, Servicio de Salud de Castilla-La Mancha, ES-45001 Toledo, Spain; 11 Centro de Salud de Sádaba, Servicio Aragonés de Salud, ES-50670 Zaragoza, Spain; 12 Centro de Salud de Sueca, Conselleria de Sanitat Universal i Salut Pública, Generalitat Valenciana, ES-46410 Valencia, Spain; 13 Centro de Salud de Huarte, Servicio Navarro de Salud, ES-31620 Pamplona, Spain; 14 Centro de Salud de Canalejas, Servicio Canario de la Salud, ES-35004 Las Palmas de Gran Canaria, Spain; 15 Departamento de Farmacología y Pediatría, Facultad de Medicina, Universidad de Málaga, ES-29010 Málaga, Spain; 16 Vaksinasjonssenter BSN, Bydel Søndre Nordstrand, Oslo kommune, NO-1252 Oslo, Norway

**Keywords:** computerized medical records systems, drug utilization, electronic prescribing, pharmacoepidemiology, polypharmacy, real-world data, Spain

## Abstract

We aimed to identify and compare medication profiles in populations with polypharmacy between 2005 and 2015. We conducted a cross-sectional study using information from the Computerized Database for Pharmacoepidemiologic Studies in Primary Care (BIFAP, Spain). We estimated the prevalence of therapeutic subgroups in all individuals 15 years of age and older with polypharmacy (≥5 drugs during ≥6 months) using the Anatomical Therapeutic Chemical classification system level 4, by sex and age group, for both calendar years. The most prescribed drugs were proton-pump inhibitors (PPIs), statins, antiplatelet agents, benzodiazepine derivatives, and angiotensin-converting enzyme inhibitors. The greatest increases between 2005 and 2015 were observed in PPIs, statins, other antidepressants, and β-blockers, while the prevalence of antiepileptics was almost tripled. We observed increases in psychotropic drugs in women and cardiovascular medications in men. By patient´s age groups, there were notable increases in antipsychotics, antidepressants, and antiepileptics (15–44 years); antidepressants, PPIs, and selective β-blockers (45–64 years); selective β-blockers, biguanides, PPIs, and statins (65–79 years); and in statins, selective β-blockers, and PPIs (80 years and older). Our results revealed important increases in the use of specific therapeutic subgroups, like PPIs, statins, and psychotropic drugs, highlighting opportunities to design and implement strategies to analyze such prescriptions’ appropriateness.

## 1. Introduction

As the average age of the population increases worldwide, public health systems should design and implement policies to adequately address the needs of an aging population [1]. As we are getting older, various chronic diseases may appear and accumulate, and we are potentially susceptible to receiving prescriptions of a variety of drugs. The co-existence of multiple chronic conditions in the same person (sometimes referred to as pluripathology or multimorbidity) and the use of many different medications at the same time (known as polypharmacy) are amongst the most important challenges for both clinicians and patients [2,3].

Patients with multiple chronic conditions and polypharmacy are more likely to experience additional morbidity and further prescriptions. The clinical management of these patients needs to implement person-centered approaches to identify potentially inappropriate medication; in some cases, polypharmacy is clinically appropriate [4]. Inappropriate (or problematic) polypharmacy and appropriate polypharmacy may result in adverse reactions, medication errors, low adherence, drug–drug and drug–disease interactions, therapeutic cascades, higher use of healthcare services, and higher mortality risk [5,6,7,8]. Various tools (such as Beers criteria and STOPP/START criteria) [9,10] and initiatives (for example, the Joint Action Chrodis-Plus of the European Union) [11] have been developed to help clinicians assess the clinical and pharmacological profile of patients with polypharmacy.

Spain has one of the highest life expectancy estimations in the world [12]. The proportion of the Spanish population aged 65 and over has been increasing during the last years: from 16.4% of the total population in 2008 to 19.4% in 2019 [13]. In a previous study, we found that, although very common in the elderly, polypharmacy affects all ages and both sexes [14]. We also showed that the prevalence of patients receiving at least five drugs has tripled in Spain during 2005–2015 (*p* < 0.001): from 2.5% in 2005 to 8.9% of the total population in 2015. Furthermore, we revealed significant differences in the prevalence of polypharmacy between sexes (with higher prevalence in women) and age groups (particularly high among individuals 80 years of age and over; 36.7% in 2015). It was reported that one in five prescriptions to elderly patients in primary care is inappropriate [15]. This observation calls for a more in-depth study of medication profiles in the elderly, especially in patients with polypharmacy. While the number of chronic medications may reveal risks of adverse outcomes, identifying the most commonly prescribed medications could help us discern possible targets for improvement; this is of particular importance considering the continuously increasing prevalence of polypharmacy in an aging population. This large-scale epidemiological study aimed to describe the medication profile of patients with polypharmacy in 2015 in Spain and assessed possible differences with data from 2005 considering sex and age group.

## 2. Materials and Methods

We conducted a retrospective cross-sectional study using information from Electronic Health Records (EHRs) registered in the Computerized Database for Pharmacoepidemiologic Studies in Primary Care (BIFAP) of the Spanish Agency of Medicines and Medical Devices (AEMPS, Madrid, Spain). The detailed profile of the BIFAP database was previously described [16,17]. BIFAP is a real-world data source with information from EHRs from patients who attended primary care facilities of the Spanish National Health System from seven regions of Spain (Aragón, Asturias, Cantabria, Castilla y León, Madrid, Murcia, and Navarra). Although there was heterogeneity in how the information was provided to BIFAP because not all participating regions share the same software, all information was harmonized into a unified data structure suitable to be used for pharmacoepidemiologic research purposes. Information collected included sociodemographic and clinical data, amongst others. All information on drug prescriptions was organized following Anatomical Therapeutic Chemical classification (ATC) codes up to the active substance level, and it included the number of packages, intended duration, and dosage. Information on the dispensation of medicines at pharmacies was being extracted from the e-prescription system; however, if not available, data from paper-based prescriptions were used. Drugs prescribed by hospital doctors, other specialists, or in the private health care setting, as well as drugs dispensed without a prescription, were not systematically registered. The main strength of BIFAP is its large sample size and its population-based nature; in 2020, BIFAP included more than 13.5 million valid and anonymous EHRs of the Spanish National Health System, corresponding to 29% of the Spanish population.

This study included information from EHRs of all patients 15 years of age and over with polypharmacy during the calendar years 2005 (i.e., 66,459 individuals; 38,062 women, 28,397 men) and 2015 (i.e., 357,347 individuals; 199,334 women, 158,013 men). Definitions and key-methodological details were described elsewhere [14]. A patient with polypharmacy was considered every person with a joint prescription of five or more courses of treatment (CT) during a minimum of a six-month period. We defined CT as the continuous prescription of medication at the chemical subgroup level of the Anatomical Therapeutic Chemical classification (ATC level 4) during at least six months; CTs cannot occur twice for the same person in one year. Cases with an interruption in the prescription (gap) greater than 30 days were not included. By constructing CTs at the ATC level 4, each course represented the chronic prescription (six months or more) of at least one active ingredient in a given year. For prescriptions upon demand, with a valid 12-month period during which the patient might use the medication if needed, a duration of 30 days for each dispensation was assigned in order to avoid mistaking these prescriptions as a 12-month continuous use. In the case of prescriptions for eye drops and topical formulations, we only included those considered as chronic (S01E, drugs for glaucoma, and miotic agents) and other prescriptions with ≥30 days of duration (D05A, antipsoriatic agents; D07A and R01A, corticosteroids, and D11AX, other dermatological products). For each patient and year, we considered only the period that maximizes the highest number of CTs. This study focused on drug prescriptions recorded in EHRs from patients attended in primary care facilities, taking into consideration intended duration but not dosage. We did not study drug dispensations because such information was not homogeneous in all participating regions in BIFAP during 2005–2015. 

We estimated the prevalence of each ATC level 4 therapeutic subgroup in patients with polypharmacy by sex and age group (15–44, 45–64, 65–79, and 80 years of age and over) in 2005 and 2015. For such calculations, the number of patients who had a prescription of an ATC level 4 subgroup during at least six months was used as a numerator. The total number of patients with polypharmacy was used as a denominator; information regarding the total number of patients with polypharmacy by age group is shown in the Supplementary Material (Table S1 for the calendar year 2015 and Table S2 for the year 2005). For the analysis of the differences between sexes or age groups, we used Pearson’s chi-squared test. Statistical analyses were conducted using SPSS version 24.0 (IBM Corp, Armonk NY, USA), and statistical significance was set at *p* < 0.05.

This research project was approved by the Scientific Committee of BIFAP, meeting its methodological and ethical requirements. BIFAP complies with the Spanish regulations for the protection of personal data. All information included in this study was anonymized.

## 3. Results

The therapeutic groups C (cardiovascular system), N (nervous system), and A (alimentary tract and metabolism) accounted for more than 70% of the CTs in 2005 and 2015. Table 1 shows the 20 most commonly prescribed CTs in patients with polypharmacy in both calendar years. The five most used chemical subgroups were: proton pump inhibitors (PPIs), HMG CoA reductase inhibitors (statins), antiplatelet agents, benzodiazepine derivatives-anxiolytics, and angiotensin-converting enzyme (ACE) inhibitors.

There were significant increases in the prevalence of prescriptions of PPIs, statins, other antidepressants, and β-blockers. The prescription of other antidepressants (mirtazapine, duloxetine, venlafaxine, and trazodone, amongst others), angiotensin II antagonists and diuretics, thyroid hormones, and α-adrenergic antagonists were almost doubled in 2015 in comparison to 2005; the prescription of other antiepileptics (gabapentin, pregabalin, lamotrigine, and topiramate, among others) was almost tripled.

Table 1 shows the 20 most frequently prescribed CTs by sex. In women, we observed a high prescription of psychotropic drugs, like anxiolytics, selective serotonin reuptake inhibitors, and other antidepressants; the prevalence of the latest increased notably. We did not observe a significant difference in the prevalence of benzodiazepine derivatives prescriptions between 2005 and 2015 in women. Anilides (such as paracetamol) were amongst the most common medications only in women, as well as it was the prescription of thyroid hormones which increased considerably in 2015 (almost doubled). In men, a cardiovascular profile was observed; in addition to antiplatelet drugs, ACE inhibitors, and sulfonamides, we also found β-blockers, dihydropyridine derivatives, and biguanides amongst the most commonly prescribed medications. In men with polypharmacy, the prescription of α-adrenergic antagonists was almost doubled.

Statistically significant differences were observed between sexes in both calendar years regarding the prevalence of the most commonly prescribed drugs in the population with polypharmacy (Table 1). An exception was the prescription of sulfonamides in 2005, with no significant difference between men and women. 

Table 2 shows the 20 most commonly prescribed CTs by age group. In the population 15–44 years, there were significant differences in the prescription of antipsychotics, other antidepressants, other antiepileptics, propionic acid derivatives, and some antipsychotics (diazepines, oxazepines, thiazepines, and oxepines). The prevalence of these antipsychotics (ATC code N05AH) was high in the population 15–44 years in 2015 (15.3%, versus 9.1% in 2005; *p* < 0.001). Another pharmacological group was not amongst the twenty most commonly prescribed drugs in 2015, but with a high prevalence in this age group (14.0% in 2015, versus 5.8% in 2005; *p* < 0.001) were the propionic acid derivatives (ATC code N05AH). In the population 45–64 years old, the prescription of other antidepressants was considerably increased, and we also observed an increase in the prescription of proton pump inhibitors and selective β-blockers. In patients 65–79 years of age, we observed an increase in the use of selective β-blockers, biguanides, proton pump inhibitors, and statins. In the population aged 80 years and over, we found higher use of statins, selective β-blockers, biguanides, other antidepressants, and proton pump inhibitors in 2015.

We observed statistically significant differences between the age groups regarding the prevalence of the 20 most commonly prescribed drugs in the population with polypharmacy (*p* ≤ 0.001) in both calendar years; the list of the most prescribed drugs and the corresponding *p* values are shown in the Supplementary Material (Table S1 for the year 2015 and Table S2 for the year 2005).

## 4. Discussion

We identified different drug prescription profiles in the Spanish population with polypharmacy between 2005 and 2015, with a different evolution regarding sex and age groups. Further, we revealed significant differences in the prevalence of the most commonly prescribed drugs between men and women, and between the age groups, in both calendar years. Our findings showed room for improving the prescription of various therapeutic groups according to the existing scientific evidence, including PPIs, statins, and benzodiazepine derivatives.

According to the Spanish National Health Survey 2011–2012, pain, allergies, locomotor, cardiovascular, and mental health problems were amongst the most frequent chronic health conditions in the Spanish population; in 2017, the profile was similar, with a higher prevalence of cardiovascular and locomotor morbidity [18]. These observations were consistent with our results, where the majority of the prescribed CTs were medications for cardiovascular, neurologic, gastroenterological, and metabolic conditions. Similar results have been reported in the literature. The IBenC Study revealed that cardiovascular and anti-ulcer drugs were used by more than one-third of home-care patients with polypharmacy in six European countries, followed by paracetamol, anti-osteoporosis drugs, and benzodiazepines [19]. A study in Italy showed that the co-prescription of one drug for peptic ulcer and gastro-esophageal reflux disease, one antithrombotic agent, and three cardiovascular medications were very common among individuals with polypharmacy [20]. Regarding pain, only paracetamol and other antiepileptics that may be indicated for this purpose were found amongst the most commonly prescribed CTs in our study population; this could suggest a possible underreporting of chronic pain medications and an on-demand prescription. A previous study estimated that the prevalence of self-medication in people over 60 years of age ranged between 20 and 60%, with analgesics and nonsteroidal anti-inflammatory drugs being the most commonly self-medicated drugs [21]. The high prevalence of antiepileptics, also used for pain and as a safe and effective method for discontinuing long-term benzodiazepine therapy in patients with generalized anxiety disorder [22,23,24], needs further research. 

It was estimated that one in ten Spanish adults aged 40–79 years has frequent symptoms of gastroesophageal reflux [25], while the incidence of peptic ulcer was one case per thousand person-years [26]. The increase in the chronic use of PPIs and their high prevalence were not consistent with the prevalence of the main health problems treated with these drugs. Possible explanations for the increased prescription of PPIs could be the frequent use of antiplatelet drugs and self-medication with propionic acid derivatives. Our results regarding the high prevalence of PPIs, especially in the elderly, were in line with findings from other studies [19,20,27,28,29], suggesting that there was considerable room for improvement regarding the chronic use of PPIs; rational deprescribing of this pharmacological group in selected patients might be a good first step to reduce the risk of adverse events and hospitalizations [30]. There were warnings about their increasing consumption worldwide in both primary and hospital care settings [31,32]: their prolonged use was related to the risk of many adverse events [33,34], so they should be used for specific indications at the minimum effective dose and during the shortest possible period [9,35]. A recently published study reported a high prevalence of legacy prescribing (despite initial appropriateness, prescribing was not discontinued after the usual effective or recommended period) of PPIs [36]; the authors hypothesized that it might represent an important issue, contributing to inappropriate polypharmacy. The same study revealed a high prevalence of co-prescriptions of PPIs and antidepressants, suggesting possible therapeutic cascades [36,37].

In our study, statins were the second most frequently prescribed therapeutic subgroup in patients with polypharmacy. The increased use of statins was well known in the literature [19,20,27,28], and it became a matter of concern [28,38]. In general, primary prevention with statins was focused on patients 40–75 years old. Primary prevention with statins in patients 75 years old and over was an area of uncertainty; a recent review concluded that clinicians should assess each case individually through person-centered approaches, considering the risks and benefits of its use in this population [39]. In addition, some side effects related to the use of statins (like myalgia and insomnia) could result in therapeutic cascades.

The joint consumption of anxiolytics and hypnotics in Spain increased by 57.4% in the period 2000–2012, from 56.7 defined daily dose per thousand inhabitants per day (DHD) in 2000 to 89.3 DHD in 2012 [40]. Our results showed that benzodiazepine derivatives were the fourth most prescribed therapeutic subgroup, the second in the population aged 15–44 years, and the fifth in patients 80 years and over. The high prevalence of benzodiazepine prescriptions was a common finding in the literature. In a recently published study among randomly selected individuals 75 years of age and older, all prescriptions of benzodiazepine derivatives were found to be inappropriate [29]. Another study reported that approximately two in three prescriptions of benzodiazepine derivatives in people 80 years old and over were inappropriate, with a high probability of adverse events [41]. In general terms, the use of benzodiazepine derivatives was not recommended for a period longer than three months [42], so these results could indicate potentially inappropriate prescriptions. Amongst the etiological factors of the benzodiazepine derivatives overuse could be the polypharmacy itself [41,43]. Benzodiazepines, antipsychotics, and opioids have significant adverse effects on the elderly and can be successfully deprescribable [30]. In our study, although opioids were not amongst the most commonly prescribed medications, we observed a three-fold increase in the prevalence of such prescriptions (ATC code N02A) between 2005 and 2015; this was a red-flag finding considering the harmful interactions of these drugs with other pharmacological groups, such as benzodiazepine derivatives [44,45,46]. According to data from the AEMPS, the use of antidepressants in Spain was tripled between 2000 and 2013 [47]. This was consistent with our results, especially in women and the population aged 15–44 years old. This notable increase suggested excessive use, despite the availability of other effective therapies and the high risk of adverse events in the elderly [48].

The prescription of thyroid hormones reached 11.5% of the population with polypharmacy, being 4.2 times higher in women than in men. A previous study in the general population 15 years of age and overestimated that the prevalence of hypothyroidism was 4.7% (lower than the suggested in the present study among patients with polypharmacy), being 4.5 times more frequent in women than in men (similar to our results) [49]. A similar comparison could be made with the original cohort of the Framingham Study, in patients 60 years of age and over with a prevalence of hypothyroidism around 4.4%, being 3.5 times higher in women [50].

The association of hyperuricemia with metabolic syndrome and increased cardiovascular risk was well known in the medical literature [2,51,52]. In this study, we found that 9.3% of the patients with polypharmacy were prescribed inhibitors of uric acid production in 2015, with a prevalence ratio of 1.6 compared to 2005. This increase could be explained, in part, by the greater use of drugs for cardiovascular diseases with aging.

The methodology we followed made it possible to identify the prescribed treatments’ duration and exclude drugs used for acute conditions. However, the number of prescribed drugs might be underestimated by calculating CTs at ATC level 4 (they may contain more than one active ingredient) and by assessing polypharmacy from January to December of each calendar year (it might shorten the estimated duration of some prescriptions). Using the ATC level 4, we avoided considering any changes of active ingredients into the same therapeutic subgroup (for example, the change from lansoprazole to omeprazole) as different CTs. The present study aimed to analyze the pharmacological therapeutic burden that the clinicians prescribe, regardless of the patients’ degree of subsequent use. No hospital or private center prescriptions were considered, no non-prescription drug dispensations, or possible self-medication.

## 5. Conclusions

This study identified differences between medication profiles in patients with polypharmacy according to sex and age group. Our data revealed important increases in the use of specific therapeutic subgroups (such as proton pump inhibitors, statins, psychotropic drugs), highlighting opportunities to design and implement specific strategies that could analyze the appropriateness of such prescriptions and the contribution of medical innovations in the clinical management of the patients, especially in women and the elderly. Proper management of a relatively small number of therapeutic subgroups could positively impact the patients’ health. For the proper management of patients with polypharmacy, it was necessary to consider the morbidity burden, sex, age, socio-economic factors, along with the patient’s involvement in decision-making. Large-scale pharmacoepidemiologic studies are essential to raise new hypotheses and implement clinical tools that could help healthcare professionals in therapeutic decision processes, especially in patients with multimorbidity and polypharmacy.

## Figures and Tables

**Table 1:** Prevalence (%) of the 20 most frequently prescribed courses of treatment (ATC level 4) in patients with polypharmacy in 2015 in both sexes and their prevalence in 2005.

ATC Level 4 ^a^		Total			Women			Men				
		2015	2005	*p*	2015	2005	*p*	2015	2005	*p*	*p*_2015_	*p*_2005_
A02BC	Proton pump inhibitors	63.3	44.3	<0.001	65.2	45.3	<0.001	60.9	43	<0.001	<0.001	<0.001
C10AA	HMG CoA ^b^ reductase inhibitors	53.5	41.7	<0.001	49.3	37.1	<0.001	58.9	47.9	<0.001	<0.001	<0.001
B01AC	Platelet aggregation inhibitors excl. heparin	37.9	40.4	<0.001	29.9	31.9	<0.001	48	51.9	<0.001	<0.001	<0.001
N05BA	Benzodiazepine derivatives	28.4	29.5	<0.001	35.8	36.2	0.091	19.1	20.4	<0.001	<0.001	<0.001
C09AA	ACE inhibitors ^c^, plain	21.6	25.7	<0.001	17.8	22.9	<0.001	26.4	29.5	<0.001	<0.001	<0.001
C03CA	Sulfonamides, plain	20.6	18.5	<0.001	21.5	18.6	<0.001	19.4	18.4	<0.001	<0.001	0.547
C07AB	Beta blocking agents, selective	20.1	12.9	<0.001	16.4	9.9	<0.001	24.7	16.8	<0.001	<0.001	<0.001
C08CA	Dihydropyridine derivatives	15.8	19.1	<0.001	14.4	17.7	<0.001	17.6	21	<0.001	<0.001	<0.001
N02BE	Anilides	15.7	13.2	<0.001	19.8	17.0	<0.001	10.6	8.1	<0.001	<0.001	<0.001
C09CA	Angiotensin II receptor blockers, plain	15.7	14.1	<0.001	15.9	14.7	<0.001	15.5	13.4	<0.001	0.002	<0.001
N06AB	Selective serotonin reuptake inhibitors	15.4	14.8	<0.001	20.4	19.6	<0.001	8.9	8.3	<0.001	<0.001	<0.001
A10BA	Biguanides	15.2	11.6	<0.001	13.2	11.2	<0.001	17.6	12.2	<0.001	<0.001	<0.001
N06AX	Other antidepressants	13.8	5.4	<0.001	17.6	6.8	<0.001	9.0	3.6	<0.001	<0.001	<0.001
C09DA	Angiotensin II receptor blockers and diuretics	13.2	6.5	<0.001	14.3	7.2	<0.001	11.8	5.6	<0.001	<0.001	<0.001
H03AA	Thyroid hormones	11.5	5.8	<0.001	17.3	8.8	<0.001	4.2	1.8	<0.001	<0.001	<0.001
G04CA	Alpha-adrenoreceptor antagonists	10.4	5.4	<0.001	0.05	0.11	0.003	23.3	12.6	<0.001	<0.001	<0.001
B01AA	Vitamin K antagonists	9.9	7.7	<0.001	8.7	7.0	<0.001	11.4	8.7	<0.001	<0.001	<0.001
A12AX	Calcium, comb. with Vit. D and/or other drugs	9.4	7.8	<0.001	14.4	11.8	<0.001	3.1	2.4	<0.001	<0.001	<0.001
M04AA	Preparations inhibiting uric acid production	9.3	5.8	<0.001	4.3	2.3	<0.001	15.5	10.6	<0.001	<0.001	<0.001
N03AX	Other antiepileptics	9.1	3.1	<0.001	10.4	3.2	<0.001	7.6	2.9	<0.001	<0.001	0.025

^a^ chemical subgroup level of the Anatomical Therapeutic Chemical Classification; ^b^ β-Hydroxy β-methylglutaryl-CoA; ^c^ angiotensin-converting-enzyme inhibitors. *p*_2015_ and *p*_2005_ represent the *p*-values of the comparisons between the sexes in 2015 and 2005, respectively. *p*-values were calculated with Pearson’s chi-squared test.

**Table 2:** Prevalence (%) of the 20 most frequently prescribed courses of treatment (ATC level 4) in patients with polypharmacy in 2015 according to age group and their prevalence in 2005.

ATC Level 4 ^a^		15–44 Years Old			45–64 Years Old			65–79 Years Old			80 Years Old and Over		
		2015	2005	*p*	2015	2005	*p*	2015	2005	*p*	2015	2005	*p*
A02BC	Proton pump inhibitors	50.0	41.6	<0.001	59.8	42.8	<0.001	62.7	44.0	<0.001	67.0	46.5	<0.001
C10AA	HMG CoA ^b^ reductase inhibitors	20.7	24.8	0.001	55.3	50.2	<0.001	60.6	45.7	<0.001	46.7	27.3	<0.001
B01AC	Platelet aggregation inhibitors excl. heparin	11.9	18.3	<0.001	32.8	37.6	<0.001	38.7	40.6	<0.001	41.6	44.5	<0.001
N05BA	Benzodiazepine derivatives	39.2	41.7	0.093	30.7	32.3	0.091	26.9	28.3	<0.001	28.0	28.2	0.551
C09AA	ACE inhibitors ^c^, plain	11.6	14.7	0.001	22.3	27.1	<0.001	21.9	25.8	<0.001	21.6	25.4	<0.001
C03CA	Sulfonamides, plain	4.7	9.4	<0.001	9.5	12.7	<0.001	16.5	16.9	0.140	32.5	27.9	<0.001
C07AB	Beta blocking agents, selective	9.0	12.8	<0.001	20.6	18.3	<0.001	22.0	13.3	<0.001	18.3	7.2	<0.001
C08CA	Dihydropyridine derivatives	5.5	11.8	<0.001	13.4	17.4	<0.001	16.7	19.8	<0.001	16.8	19.8	<0.001
N02BE	Anilides	10.5	7.2	<0.001	10.9	8.4	<0.001	13.9	12.8	<0.001	21.0	18.6	<0.001
C09CA	Angiotensin II receptor blockers, plain	6.7	8.6	0.016	14.0	14.2	0.504	16.5	15.0	<0.001	16.5	12.8	<0.001
N06AB	Selective serotonin reuptake inhibitors	24.0	25.3	0.296	17.2	17.9	0.048	13.9	13.2	0.001	15.4	14.4	0.001
A10BA	Biguanides	6.5	7.0	0.511	17.0	17.1	0.767	17.8	12.4	<0.001	11.7	5.7	<0.001
N06AX	Other antidepressants	22.4	12.4	<0.001	15.9	6.8	<0.001	11.3	4.6	<0.001	14.8	5.2	<0.001
C09DA	Angiotensin II receptor blockers and diuretics	2.1	2.2	0.829	9.8	6.6	<0.001	15.2	7.3	<0.001	13.6	5.2	<0.001
H03AA	Thyroid hormones	13.5	6.0	<0.001	13.6	7.7	<0.001	12.3	5.8	<0.001	9.3	4.1	<0.001
G04CA	Alpha-adrenoreceptor antagonists	0.6	1.0	0.066	4.2	2.7	<0.001	11.9	6.6	<0.001	12.8	5.9	<0.001
B01AA	Vitamin K antagonists	2.4	2.0	0.461	4.9	5.6	<0.001	9.9	8.5	<0.001	13.4	8.4	<0.001
A12AX	Calcium, comb. with Vit. D and/or other drugs	5.1	5.4	0.681	7.0	7.2	0.446	9.9	8.4	<0.001	10.4	7.2	<0.001
M04AA	Preparations inhibiting uric acid production	3.2	4.2	0.091	7.5	6.1	<0.001	10.0	6.1	<0.001	10.0	5.1	<0.001
N03AX	Other antiepileptics	25.1	14.9	<0.001	12.9	4.6	<0.001	8.0	2.6	<0.001	7.2	1.9	<0.001

^a^ chemical subgroup level of the Anatomical Therapeutic Chemical Classification; ^b^ β-Hydroxy β-methylglutaryl-CoA; ^c^ angiotensin-converting-enzyme inhibitors. *p* values were calculated with the Pearson’s chi-squared test.

## Data Availability

The data used in this study cannot be publicly shared, because of restrictions imposed by the Computerized Database for Pharmacoepidemiologic Studies in Primary Care (BIFAP) of the Spanish Agency of Medicines and Medical Devices (AEMPS, Madrid, Spain). Researchers who wish to access the data should get in touch with BIFAP.

## Supplementary Materials

The following are available online at https://www.mdpi.com/article/10.3390/ijerph18094754/s1, Table S1: Prevalence (%) of the 20 most frequently prescribed courses of treatment (ATC level 4) in pa-tients with polypharmacy in 2015 according to age group. Table S2: Prevalence (%) of the 20 most frequently prescribed courses of treatment (ATC level 4) in patients with polypharmacy in 2005 according to age group.
